# Acute Pancreatitis and Splenic Infarction as Initial Symptoms of a Male Patient With SLE: A Rare Case Report

**DOI:** 10.1002/ccr3.73038

**Published:** 2026-06-26

**Authors:** Jian‐mei Gong, Xu Jun

**Affiliations:** ^1^ Clinical Medicine Guizhou Medical University Guiyang City Guizhou Province China; ^2^ Department of Nephrology Affiliated Hospital of Guizhou Medical University Guiyang City Guizhou Province China

**Keywords:** acute pancreatitis, immunosuppressants, lupus nephritis, splenic infarction, systemic lupus erythematosus

## Abstract

Systemic lupus erythematosus (SLE) can rarely present as concurrent acute pancreatitis (AP), splenic infarction, and renal failure. This case demonstrates that prompt diagnosis and immunosuppression are crucial for reversing organ damage—even in male patients, expanding the known clinical spectrum of this disease.

## 1 Introduction

Systemic lupus erythematosus (SLE) is a multisystem autoimmune disease with a chronic course and variable manifestations. SLE affects roughly 20 to 150 people per 100,000 [1], with a higher incidence in women (a female‐to‐male ratio of 6:1 to 10:1) [2]. The presence of multiple autoantibodies, which leads to the formation and deposition of immune complexes (ICs), could be considered a disease hallmark. SLE can virtually affect any organ system, with a wide heterogeneity in terms of clinical manifestations, requiring personalized treatments [3]. Acute pancreatitis (AP) and splenic infarction (SI) are rare, life‐threatening complications of SLE. These patients are often present with abdominal pain as the first symptom, making diagnosis difficult. This requires clinicians to strengthen their awareness, diagnosis, and treatment of the disease. We present a report of a 50‐year‐old male patient who initially presented with abdominal pain as the predominant complaint, accompanied by fever, diarrhea, and oliguria. Based on clinical and radiological findings, he was diagnosed with acute necrotizing pancreatitis, renal failure, and splenic infarction. However, his condition failed to improve significantly following targeted treatment for these initial diagnoses. Subsequent immunological investigations and renal pathological examination confirmed the diagnosis of SLE. Upon definitive diagnosis, the patient received high‐dose glucocorticoids combined with immunosuppressants (hydroxychloroquine and belimumab). The patient exhibited rapid resolution of gastrointestinal symptoms alongside improved renal function, with follow‐up imaging confirming resolution of splenic infarction and subsidence of pancreatic inflammation.

## 2 Case History

A 50‐year‐old male presented with a 20‐day history of persistent abdominal pain. During the acute phase, the patient exhibited a high‐grade fever (peak temperature: 40°C) and frequent diarrhea (approximately once per hour). Notably, he reported oliguria with a daily urine output of approximately 200 mL. There was an absence of skin lesions, photosensitivity, alopecia, or arthralgia. There was no personal or family history of autoimmune disorders.

## 3 Methods

**FIGURE 1:**
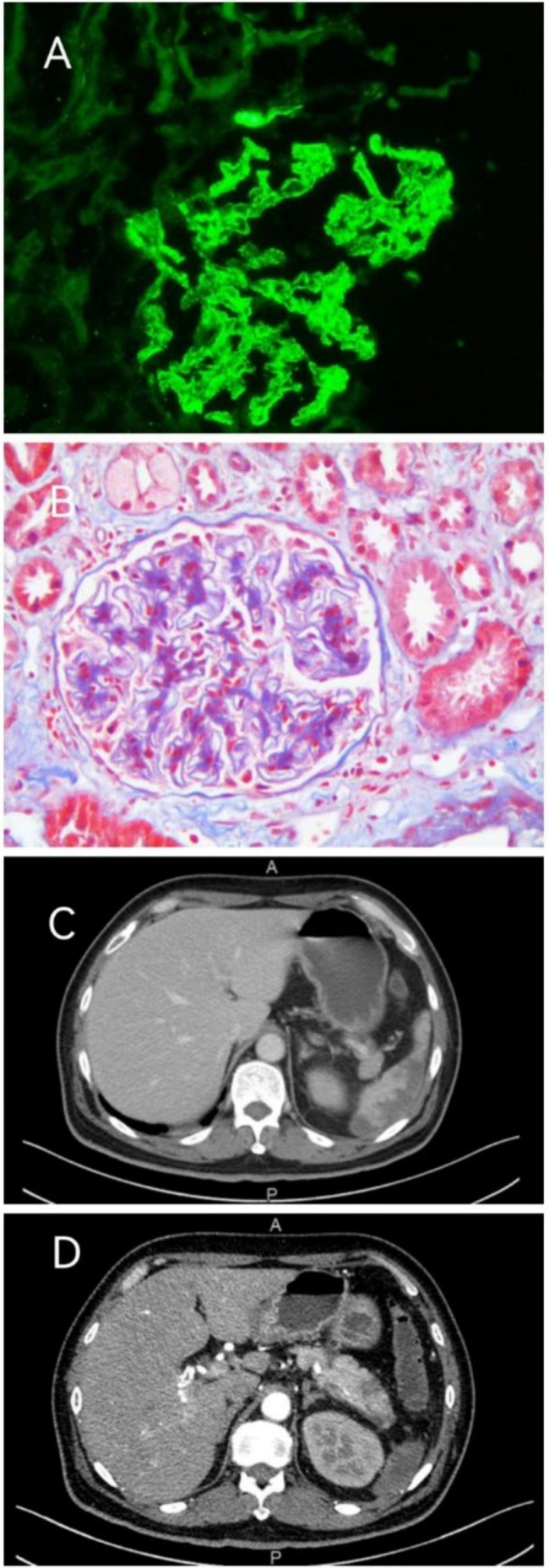
Pathological and contrast‐enhanced CT images from a male SLE patient with initial manifestations of splenic infarction and acute necrotizing pancreatitis. (A) Glomerular granular immune complex deposition under immunofluorescence; (B) Light microscopy identifies ISN/RPS class V membranous lupus nephritis combined with ischemic tubulointerstitial injury; (C) Patchy hypodense infarct lesions in the spleen on initial CT; (D) Heterogeneous pancreatic enhancement and peripancreatic exudates indicative of necrotizing pancreatitis on abdominal CT.

The patient was found to have a severe infection (WBC 14.56 × 10^9^/L with 91.4% neutrophils, PCT 2.33 ng/mL, IL‐6: 30.11 pg/mL, high‐sensitivity CRP 156.61 mg/L), abnormal liver function (ALT 151.00 U/L, AST 198.80 U/L, ALB 26.56 g/L), renal failure (CREA 726 μmol/L, eGFR 9.47 mL/min/1.73 m^2^), elevated urinary protein (micro total protein 2207.77 mg/24 h), and coagulation dysfunction (FIB: 5.71 g/L, D‐dimer 12.02 μg/mL, FDP: 49.55 μg/mL). Contrast‐enhanced computed tomography (CT) of the entire abdomen revealed: (1) Multiple patchy nonenhancing hypo‐dense lesions in the spleen, likely representing splenic infarction (Figure 1C). (2) Abnormal enhancement in the body and tail of the pancreas: Acute necrotizing pancreatitis is suspected, with surrounding exudative changes (Figure 1D). Based on the above clinical manifestations, laboratory, and imaging findings, the patient demonstrates multi‐system damage. An immune‐mediated disease is suspected to underlie the multi‐organ involvement. Therefore, further immunological testing (IgG 18.10 g/L, IgM 0.236 g/L, and C3 0.457 g/L; anti‐nRNP antibody was strongly positive, anti‐Sm antibody was strongly positive, antiphospholipid antibodies (aPL) were negative, anti‐double‐stranded DNA antibody was negative; direct Coombs test was positive) and renal biopsy were performed for definitive diagnosis. Results of kidney needle biopsy: membranous lupus nephritis (ISN/RPS classification: type V) with ischemic renal injury; subacute tubulointerstitial injury (Figure 1A,B).

Based on the clinical manifestations and diagnostic findings, the patient was definitively diagnosed with SLE presenting with multi‐organ involvement, including AP, splenic infarction, and renal failure. The patient was treated with the following regimen: methylprednisolone pulse therapy (200 mg/day for 5 days), followed by a maintenance dose of 50 mg/day; intravenous immunoglobulin (20 g/day for 3 days); belimumab (600 mg per dose, administered twice at two‐week intervals); hydroxychloroquine (400 mg/day); antimicrobial therapy; hemodialysis; anticoagulation; and electrolyte balance maintenance.

## 4 Conclusion

Following the above interventions, the patient exhibited significant improvement in gastrointestinal symptoms, restoration of normal urinary output, and normalization of body temperature. Laboratory parameters demonstrated marked improvement: serum creatinine decreased from 726 μmol/L to 105 μmol/L, and D‐dimer levels returned to the normal range. Repeat CT scan revealed reduced pancreatic swelling and resolution of splenic infarction. After discharge, the patient continued belimumab therapy (600 mg per dose, administered approximately every 30 days). Follow‐up evaluations showed a reduction in 24‐h urine protein excretion from 2207.77 mg/24 h to 5.66 mg/24 h, with serum creatinine further decreasing to 80.5 μmol/L. No disease recurrence was observed during follow‐up.

## 5 Discussion

AP represents an uncommon yet potentially life‐threatening complication of SLE. A seminal study by Nesher et al. demonstrated AP occurrence in 28.8% (21/73) of patients during SLE exacerbations, with abdominal pain serving as the predominant clinical manifestation (88%–93% of cases) [4]. The pathogenesis of SLE‐associated AP involves multifactorial mechanisms, including: (1) Microvascular injury: Vasculitis, immune complex deposition, and arteriolar occlusion; (2) Immune dysregulation: Autoantibody‐mediated tissue damage and aberrant T‐cell responses; (3) Iatrogenic factors: Drug‐induced pancreatic toxicity (e.g., corticosteroids, immunosuppressants) [5]. The diagnosis of AP relies heavily on advanced imaging modalities, particularly contrast‐enhanced computed tomography (CECT) and magnetic resonance imaging (MRI). However, in patients with SLE, the use of iodinated contrast agents in CECT raises clinical concerns due to their nephrotoxic potential, especially in those with preexisting lupus nephritis. This dilemma often results in delayed or underdiagnosis of AP within this population. CT better characterizes complications such as pancreatic edema, necrosis, and peripancreatic fluid collections. Severity of necrosis in CT can predict outcome of AP [6]. MRI offers notable advantages, including the absence of ionizing radiation and the ability to obtain valuable tissue information without contrast administration, thereby reducing the risk of contrast‐induced nephrotoxicity. In the diagnosis of AP, MRI outperforms CT [7]. Noncontrast MRI can serve as a first‐line imaging option to avoid contrast‐induced nephropathy. While certain immunosuppressive agents—including corticosteroids, azathioprine, and cyclosporine—have been implicated in drug‐induced pancreatitis based on case reports [8], this patient's clinical course provides countervailing insights. He developed AP prior to any immunosuppressive therapy, and the subsequent initiation of high‐dose glucocorticoids correlated with rapid resolution of pancreatic inflammation. This paradoxical response suggests that glucocorticoids may exert therapeutic rather than pathogenic effects in SLE‐related AP, potentially by mitigating lupus‐driven microvascular injury and systemic inflammatory responses.

Splenic involvement in SLE manifests most commonly as periarterial fibrosis or “onion‐skin” vascular lesions, reflecting chronic immune‐mediated vasculopathy [9]. As of February 7, 2023, only 36 cases of SLE‐associated SI have been documented in the literature, underscoring its rarity [10]. Approximately half of the reported patients had no abdominal manifestations, and the presence of SI was found incidentally during screening. Therefore, SI is easily overlooked. Notably, SI frequently coincides with multiorgan infarction (kidneys, brain, lungs, intestines), necessitating comprehensive vascular evaluation upon diagnosis [10]. Local thrombosis, vascular occlusion, vasculitis, and perivascular inflammation impair the splenic circulation and lead to SI [11]. Early intervention with glucocorticoids, immunosuppressants, and anticoagulation achieves rapid resolution.

The constellation of multi‐system involvement (pancreatic necrosis, splenic infarction, renal failure, and coagulopathy) in the absence of classic SLE manifestations underscores the diagnostic complexity. This case highlights the imperative for early immunological and histological evaluation in patients with atypical multi‐organ dysfunction. A hypercoagulable state and vasculitis are common clinical features of SLE. Patients with SLE are often complicated by vasculitis, which is strongly associated with organ infarction. SLE‐induced small‐vessel vasculitis can damage the vascular endothelium of the spleen and pancreas, leading to vascular stenosis, occlusion, and subsequent infarction and inflammation. Although aPL were negative, SLE itself can trigger a hypercoagulable state through immune complex deposition, activation of the complement system, and abnormal platelet function, which further promotes thrombosis and tissue ischemia [12]. Shared mechanisms between aPL and other autoantibodies may account for the increased thrombosis risk in aPL‐negative SLE patients [12]. In the study by Li Wang, only 41.2% (7/17) of systemic SLE patients with thrombotic events were positive for aPL [13]. Timely use of corticosteroids and immunosuppressants can significantly improve the condition and reduce mortality. This requires clinicians to strengthen their understanding of the multiple organ damage caused by SLE, and early and accurate diagnosis and treatment can greatly improve the prognosis of patients.

## Author Contributions

**Jian‐mei Gong:** formal analysis, investigation, methodology, visualization, writing – original draft, writing – review and editing. **Xu Jun:** conceptualization, data curation, funding acquisition, methodology, project administration, resources, software, supervision, validation.

## Funding

The authors have nothing to report.

## Consent

The patient has provided written informed consent for the publication of this case report. A copy of the consent form has been submitted alongside the manuscript files.

## Data Availability

The data used in this article are available upon request from the authors.
